# Simultaneous Bilateral Transient Osteoporosis of the Hip without Pregnancy

**DOI:** 10.1155/2016/8491461

**Published:** 2016-08-28

**Authors:** Yasuaki Okada, Sachiyuki Tsukada, Masayoshi Saito, Atsushi Tasaki

**Affiliations:** Department of Orthopaedic Surgery, St. Luke's International Hospital, 9-1 Akashi-cho, Chuo-ku, Tokyo 104-8560, Japan

## Abstract

Transient osteoporosis of the hip (TOH) is a rare disorder characterized by acute severe coxalgia and temporary osteopenia in the proximal femur. Although most cases were unilateral or staged bilateral TOH, some authors reported that the pregnant patients simultaneously had TOH in their bilateral hips. However, there has been no report of simultaneous bilateral TOH in the patient without pregnancy. A 25-year-old Japanese woman without pregnancy had acute simultaneous bilateral hip pain. Plain X-ray of the bilateral hips did not show a periarticular osteopenia. However, magnetic resonance image obtained one week after the onset demonstrated increased T2-weighted signal intensity and decreased T1-weighted signal intensity in the bilateral femoral heads. She was treated conservatively, and follow-up magnetic resonance image at seven weeks after the onset returned to normal bone marrow signal intensity. Her bilateral coxalgia subsided gradually. At one year after the onset, she had no sign of symptomatic flair. Our experience with this case indicates that recognizing the possibility of simultaneous bilateral TOH is important unless the patient is pregnant, and magnetic resonance image is predictable test to make a diagnosis of TOH, even in the absence of abnormal finding on plain X-ray.

## 1. Introduction

Transient osteoporosis of the hip (TOH) is a rare condition that causes temporary bone loss in the proximal femur and sudden-onset severe hip pain [1, 2]. Most previously reported cases of TOH affected a unilateral hip joint or staged bilateral hip joints; however, some authors reported cases in which pregnant female patients simultaneously had TOH in bilateral hips [3–6]. To our knowledge, there have been no reports on patients without pregnancy but with simultaneous bilateral TOH. Although the pathophysiology of TOH has not been clarified, the cause of simultaneous bilateral TOH has been believed to be associated with pregnancy based on previous case reports [3–6].

We report a 25-year-old woman without pregnancy who had bilateral simultaneous hip TOH. The patient was informed that data concerning the case would be submitted for publication and provided consent for study.

## 2. Case Report

A 25-year-old Japanese nonpregnant woman without a significant medical history developed acute bilateral hip pain that progressively increased over the span of a few days. Her occupation was radiology technologist, and she has not been physically active on a regular basis. The onset of hip pain was not associated with trauma, and the patient had no other predisposing factors for osteonecrosis. She was unable to bear weight and walked with a limp. When walking, her right and left hip pain score evaluated via numeric rating scale were eight and six, respectively.

On physical examination, she was 160 cm tall and weighed 53.6 kg with a body mass index of 20.9 kg/m^2^. Bilateral hips were positive on the Patrick test. Both anterior and posterior impingement tests were negative for bilateral hips. The following ranges of motion were obtained: flexion, right 90 degrees/left 110 degrees; extension, right 10 degrees/left 20 degrees; abduction, right 20 degrees/left 30 degrees; adduction, right 30 degrees/left 40 degrees; internal rotation, right 20 degrees/left 30 degrees; and external rotation, right 30 degrees/left 40 degrees.

She was afebrile. Her blood tests for hematology, biochemistry, coagulation, erythrocyte sedimentation rate, endocrine, rheumatoid arthritis, and bone metabolism were unremarkable. Calcium was 9.4 mEq/L (normal, 8.4 to 10.2 mEq/L), intact parathyroid hormone was 32 pg/mL (normal, 10.0 to 65.0 pg/mL), and 25-hydroxyvitamin D measured with a DiaSorin radioimmunoassay was 16.4 ng/mL (normal, 9.0 to 37.6 ng/mL).

**Figure 1:**
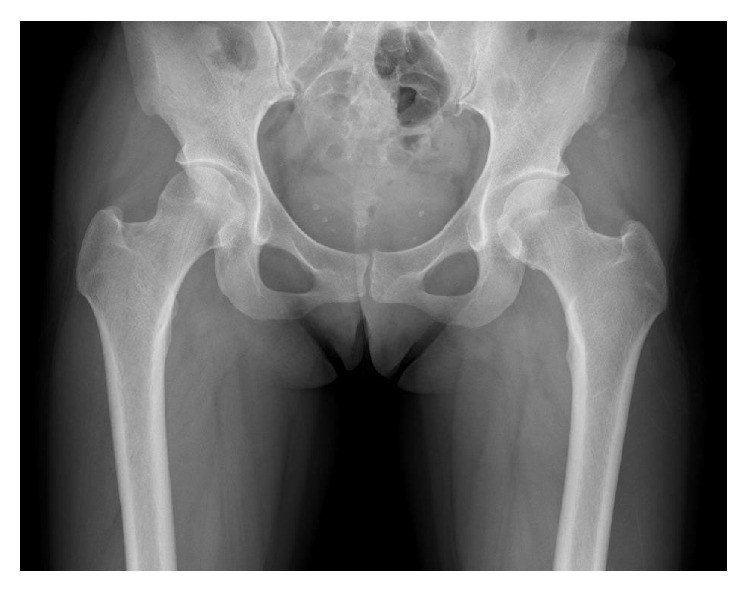
Anteroposterior X-ray of pelvis one week after symptom onset. No osteopenia.

**Figure 2:**
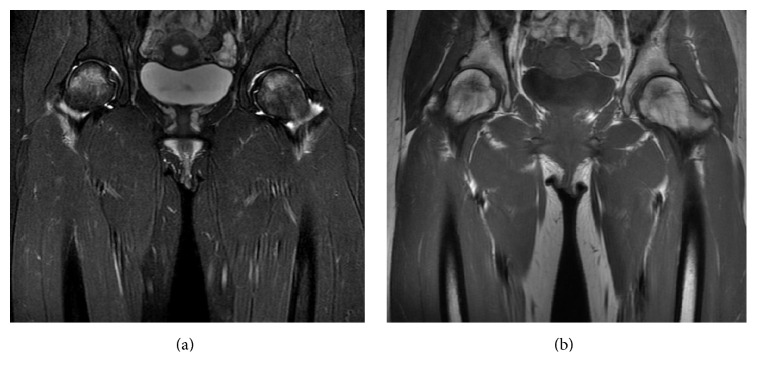
Magnetic resonance images of pelvis one week after symptom onset. (a) Coronal T2-weighted magnetic resonance image. Increased signal uptake within the femoral heads and necks bilaterally. There were no signs of osteonecrosis. (b) Coronal T1-weighted magnetic resonance image. Decreased signal uptake within the femoral heads and necks bilaterally.

**Figure 3:**
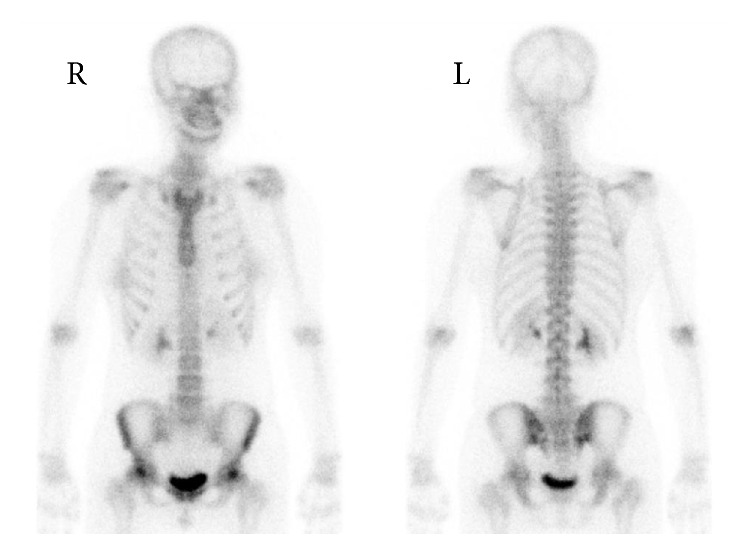
Bone scintigraphy of pelvis one month after symptom onset. Increased signal uptake within the femoral heads and necks bilaterally.

Plain anteroposterior view X-ray of the pelvis and hips one week after symptom onset did not show an obvious appearance of osteopenia or diffuse thinning of the cortex of the bilateral femoral head and neck (Figure 1). There was no evidence of dysplasia of the bilateral hips (Figure 1). The right hip had a center-edge angle of 27.9 degrees (normal in Japanese women, 27.0 to 34.0 degrees), the sharp angle was 45.3 degrees (normal in Japanese women, 34.0 to 42.0 degrees), and the acetabular head index was 79% (normal in Japanese women, 80 to 89%). The left hip had a center-edge angle of 27.3 degrees and sharp angle of 49.7 degrees. The acetabular head index was 83.7%. A magnetic resonance image obtained on the same day, however, demonstrated increased T2-weighted signal intensity and decreased T1-weighted signal intensity in the bilateral femoral heads consistent with bone marrow edema, and there were no findings to suggest osteonecrosis (Figure 2). Bone mineral density was measured with dual energy X-ray absorptiometry at nine days after onset: the *Z*-sore of the left femoral neck was −0.5 and that of lumbar spine was 0.3. A 740 MBq Tc 99-labeled methyl diphosphonate bone scintigraphy to distinguish neoplasm showed increased uptake only in bilateral femoral heads (Figure 3).

**Figure 4:**
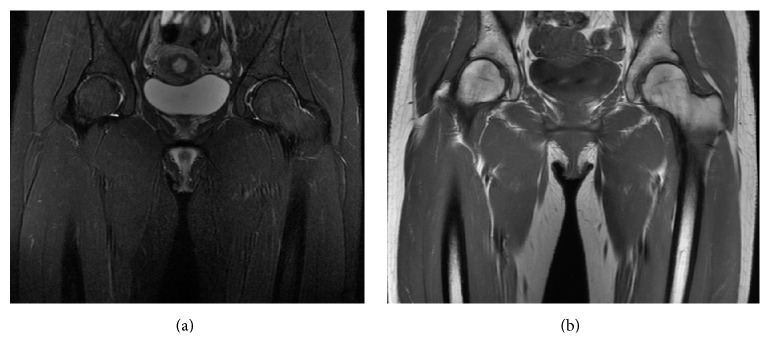
Magnetic resonance images of pelvis seven weeks after symptom onset. (a) Coronal T2-weighted magnetic resonance image. Normal signal uptake within the femoral heads and necks bilaterally. (b) Coronal T1-weighted magnetic resonance image. Normal signal uptake within the femoral heads and necks bilaterally.

We recommended conservative treatment with analgesics and protected weight bearing. At first, she remained non-weight-bearing with bed rest and a wheel chair and took an oral nonsteroidal anti-inflammatory drug (25 mg of diclofenac [Voltaren]; Novartis Pharmaceuticals Japan, Tokyo, Japan) three times per day. Despite continued symptoms, magnetic resonance image seven weeks after onset demonstrated a return of normal bone marrow signal intensity in the bilateral femoral heads (Figure 4). Her symptoms gradually resolved eight weeks after onset. The results of these tests and clinical course led us to make a tentative diagnosis of TOH.

Pain and range of motion in the left hip improved earlier than those in the right hip. Partial weight bearing with crutches was allowed eight weeks after onset. She was discharged from the hospital with crutches and took no analgesics two months after onset. The left hip was fully improved with a normal range of motion three months after onset, and full weight bearing without pain was possible. By four months, the range of motion in bilateral hips had completely recovered. At this point, she was permitted to gradually return to her job. The intensity was gradually increased, and her return to full activity was permitted at nine months after onset.

She was subsequently followed up over a period of 12 months. Follow-up X-ray and magnetic resonance image were normal. She was able to return to her normal level of activity.

## 3. Discussion

This case suggested two clinical issues. First, a patient without pregnancy may have simultaneous bilateral TOH. Second, magnetic resonance image is useful in detecting this disorder even in the absence of abnormal X-ray and laboratory test findings.

Our case report revealed that a patient without pregnancy could have simultaneous bilateral TOH. TOH usually affects pregnant women in the third trimester or middle-aged men [2, 7]. All patients of previous reports involving simultaneous bilateral TOH were pregnant women [3–6]. Although the cause of TOH of young woman remains unclear, Rajak and Camilleri noted a clear association between pregnancy and TOH [8]. Investigators advocated that the etiology of the TOH involved obturator nerve compressions or local vascular blocks by the fetus, defects in fibrinolysis due to pregnancy causing ischemia in the bone, and deficiencies in bone metabolism such as vitamin D deficiency [7]. In previous simultaneous bilateral TOH cases, Axt-Fliedner et al. reported a pregnant woman with functional recovery after the termination of pregnancy via cesarean section due to limited motion in both hips [4]. Willis-Owen et al. reported a pregnant woman with simultaneous bilateral TOH following a femoral neck fracture [5]. Emami et al. also reported a pregnant woman who had simultaneous bilateral TOH following femoral neck fracture and noted that the mechanism of TOH was considered to be a microvascular disorder leading to tissue ischemia [6]. We believe that our patient (without pregnancy) could provide the new clue in determining the cause of bilateral simultaneous TOH.

Magnetic resonance imaging was useful in detecting TOH when plain X-ray showed no abnormalities. Although plain X-ray is believed to typically show pronounced demineralization and the loss of a trabecular pattern in the femoral head and neck [1, 9], no radiographic evidence of demineralization may exist during early symptom onset [10]. Our case had no remarkable plain X-ray findings at one week after symptom onset, and some investigators reported normal plain X-ray findings in the early stage of TOH [11, 12]. Magnetic resonance imaging shows diffuse bone marrow edema with increased signal on T2-weighted images and decreased signal on T1-weighted images in TOH [13]. Characteristic findings of TOH in magnetic resonance imaging include the complete resolution of abnormal signal intensity over several weeks [14]. Our report supported the utility of repeated magnetic resonance imaging in a patient suspected to be affected by TOH.

Several investigators recommend distinguishing TOH from transient bone marrow edema syndrome [14–16]. Hayes et al. advocated that the term “transient bone marrow edema syndrome” should be used for patients in whom the bone marrow edema pattern was not accompanied by radiographic evidence of osteopenia [14]. Our case fulfilled the criteria of Hayes et al. for transient bone marrow edema syndrome. However, we believe that distinguishing between the two terms is rarely useful in clinical practice because it may not impact the therapeutic strategy.

Bone mineral density measured at nine days after onset remained within the normal range in this case. Several reports suggested the relationship between TOH and low bone mineral density [17, 18]. Because bone mineral density changes with time in TOH [18], single-point measurement may cause the normal finding of bone mineral density in this case.

TOH is a rare disorder that cannot be easily diagnosed. Clinicians should be aware of the possibility of simultaneous bilateral hip pain caused by TOH unless the patient is pregnant. We recommend advanced imaging, especially magnetic resonance imaging, if the patient is suspected to be affected by TOH without abnormal findings on plain X-ray.

## 4. Conclusions

To the best of our knowledge, this is the first case report of simultaneous bilateral TOH in a patient without pregnancy. Notably, unless the patient is pregnant, the patient could be affected by simultaneous bilateral TOH. If the patient is suspected to be affected by TOH without clear abnormal findings via plain X-ray, magnetic resonance imaging is recommended for diagnosis and in monitoring disease progression.
